# The impact of cognitive-motor interference on balance and gait in hearing-impaired older adults: a systematic review

**DOI:** 10.1186/s11556-024-00350-x

**Published:** 2024-06-24

**Authors:** Anna Wunderlich, Bettina Wollesen, Janek Asamoah, Kim Delbaere, Karen Li

**Affiliations:** 1 Technische Universität Berlin, Chair of Biopsychology and Neuroergonomics, Berlin, Germany; 2 Faculty of Psychology and Human Movement Science, Institute for Human Movement Science, Universität Hamburg, Hamburg, Germany; 3 Faculty of Psychology and Human Movement Science, Institute for Human Movement Science, Universität Hamburg, Hamburg, Germany; 4 Neuroscience Research Australia (NeuRA) Falls, Balance and Injury Research Centre, Sydney, NSW Australia; 5 Population Health, Faculty of Medicine and Health, University of New South Wales, Sydney, NSW Australia; 6 Department of Psychology, Concordia University, Montreal, Canada

**Keywords:** Cognitive-motor interference, Balance, Gait, Hearing loss

## Abstract

**Background:**

Hearing impairments are a rising burden in our aging society. Hearing loss is associated with reduced cognitive performance as well as decrements in balance and gait. Therefore, impaired hearing affects also dual tasking (DT). The aim of this review is to summarize the evidence for DT performance decrements of older adults with hearing impairments during maintaining balance or walking.

**Methods:**

The systematic literature research according to PRISMA guidelines was conducted using MEDLINE, APA Psych-Info, and Web of Science. Inclusion criteria were: Independent living older people ≥ 60 years with hearing impairments, use of a DT paradigm to test hearing impaired older adults within a balance or walking condition.

**Results:**

*N* = 57 studies were found within the databases. Eight studies were included (*N* = 456 participants (58% women), including *n* = 200 older hearing-impaired persons with different levels of hearing loss). Most of the included studies oriented their inclusion criteria for hearing-impairments at thresholds for mild hearing loss with Pure Tone Average (0.5-4 kHz) ≥ 25 and < 40 dB. Three of the studies focused on DT balance performance and five used DT walking comparing participants with and without hearing loss. For DT balance and gait performance, higher decrements for the hearing-impaired group were observed compared to healthy older adults. Performance decrements were accompanied by reduced compensatory strategies in balance performance.

**Conclusion:**

More pronounced decrements in DT performance were observed for participants with hearing impairments compared to those without. This implies that hearing-impaired older adults might need specific interventions to reduce the cognitive-motor interference (CMI) to maintain balance control or walking stability in daily situations that require managing of cognitive and motor tasks simultaneously. However, taking all results into account the underlying mechanisms of CMI for this target group needs to be further examined.

**Trial registration:**

This review was registered at Prospero with the ID CRD42022340232.

**Supplementary Information:**

The online version contains supplementary material available at 10.1186/s11556-024-00350-x.

## Introduction

Age-related hearing impairment is a prevalent condition affecting nearly every second person over the age of 65 years [1]. It represents a global health challenge, with about 5% of the world population affected, a number projected to rise to 8% by 2050 [2]. Hearing impairment can impact social and emotional well-being [3] and limit capacity for daily activities and physical functioning [4–7]. As the world’s population continues to age, hearing impairment should therefore be considered a worldwide public health burden.

Hearing impairment has been associated with poorer cognitive performance [8–11], which may be attributed to age-related neural degeneration, sensory deprivation and reduced cognitive reserve. This can result in hearing impaired adults requiring additional cognitive resources for auditory processing, leaving fewer resources available for other cognitive processes [10]. Moreover, age-related changes in the auditory system lead to higher pure-tone detection thresholds and supra-threshold auditory difficulties [12] can make auditory processing more cognitively demanding [13]. Hearing loss and auditory dysfunction have also been associated with an increased risk of dementia [11, 14].

Previous research has linked hearing loss with balance impairments [15, 16], subjective walking limitations [17], reduced physical fitness [5], and increased frailty [18]. The severity of the hearing impairment has been connected to decrements in spatio-temporal gait parameters and falls [19]. Age-related changes in the vestibular system and proprioceptive functions further contribute to balance problems in hearing-impaired older adults [20], due to reduced sensitivity and integration of sensory information. These changes result in less efficient compensatory movements, affecting balance control during upright walking. Central problems in vestibular perception concern the vestibulo-ocular reflex and the vestibulo-spinal reflex, both of which are responsible for head position and eye movement control during upright walking [21]. The reason for this is attributed to changes in the hair and nerve cells in the vestibular apparatus, which subsequently react less sensitively to information, absorbing and transmitting it to a limited extent [21]. Similar losses are also assumed for proprioception. Balance problems can therefore be attributed primarily to the lack of optimal integration between the visual, auditory, vestibular and proprioceptive sensory information [22]. Accordingly, various aspects of the aging process result in less reliable sensory information and less accurate integration of information. According to Lindenberger [22], this leads to less efficient compensatory movements that serve to maintain balance. As a result, e.g., the postural sway or sway velocity is increased and reduces the margins of stability [23].

Additionally, walking in daily life often involves multitasking, such as crossing the street while reading signs and/or monitoring traffic [24]. This means that in everyday life, balancing or walking can be described as a dual-task (DT) activity [24], which is associated with decreased walking and balance performance, potentially increasing the risk of falling. Reduced walking performance is characterized by increased variability in foot placement, increased double support time, as well as reduced step length and velocity [25–28]. Hearing impairment further affects gait parameters (speed, phase and rhythm) under dual-task conditions, independent of age and comorbidities [29]. The association of hearing impairment and mobility decline can be attributed to competition for limited cognitive resources [30]. Finally, research indicates that changes of the sensory information results in greater declines in postural control for older adults compared to younger adults [31, 32]. When auditory challenges are introduced during balancing or walking tasks, there is an increased competition for cognitive capacity [33].

Despite these associations, the interaction between age-related hearing impairment and cognitive-motor interference on balance and walking performance is poorly understood. However, detecting these aspects is highly relevant to conduct tailored training interventions for this target group.

Therefore, the specific research question of this literature review was to understand how dual-task performance affects gait or balance parameters in older adults with hearing impairments. Additionally, the review will describe how hearing loss has been defined across studies, the types of DT combinations used in measurements (e.g. task complexity, stimulus–response condition), and the identified interaction between the severity of hearing loss and complexity of the balance and walking tasks.

We are aware that the methodological differences between studies make it difficult to answer the research question conclusively. However, we expect that older adults with hearing loss consistently show decreased dual-task performance compared to healthy controls.

The overall goal is to derive best practice recommendations for future cognitive-motor DT studies for this target group.

## Methods

### Search strategy and selection criteria

Three databases were systematically searched by using OvidSP to search in Medline (1946 to 2022, Week 30, APA PsycINFO (1806 to 2022, Week 30) as well as Web of Science (25.07.2022). The search strategy was to use combinations of the following key terms (Table 1).

**Table 1:** Search overview

**Search stage**	**Papers retained**		
	**Medline**	**APA PsycInfo**	**Web of Science**
1. "Age" or "old$" or "elder$" or "aged" or "advanced age" or "senior$" or "geriatric$" or "eldest" or "aging" or "geronic"	8,297,766	1,416,280	5,231,007
2. "corresponding task$" or "coupled task$" or "dual task$" or "dual task paradigm$" or "secondary task" or "conflicting task" or "Dual-task cost$"	6,510	4,916	2,985
3. "Gait" or "walking" or "Step" or "stride" or "balance" or "postural sway" or "EMG" or "COP displacement" or "center or pressure" or "kinematics" or "Cadence" or "Double support$" or "stance phase" or "swing phase"	8,240,436	111,512	1,907
4. "hearing loss" or "hearing impaired" or "hearing impairment" or "pure tone audio$" or "pure tone"	89,344	14,817	20
5.Combination of all four (1 & 2 & 3 & 4)	36	5	16
Assessment based on reading the whole paper	7	4	7
Overall included studies: 8			

Two reviewers independently searched within titles and abstracts to identify all potentially eligible studies meeting the inclusion criteria. In addition, the reference lists of the retrieved articles that fulfilled the inclusion criteria were searched manually.

### Eligibility criteria

This review focused on older adults with hearing-impairment (Pure Tone Average (PTA) 2–4 kHz > 19.5 dB) and its association with cognitive-motor interference, balance and gait performance. With regards to the classification of participants according to their hearing ability, we chose the World Health Organization (WHO; [34]) definition as our main reference. The WHO proposes in the report different grades of hearing loss by using certain thresholds of the minimum sound intensity that an ear can detect as an average of values at 500, 1000, 2000, 4000 Hz in the better hearing ear. The specified thresholds are: Mild hearing impairment (20–34 dB), moderate (34–49 dB), moderate severe (50–64 dB), severe (65–79 dB), profound (80–94 dB), and complete hearing loss (> 95 dB) [34]. However, we allow for different approaches to classify participants with respect to their hearing ability and report this classification as the first outcome.

Therefore, the inclusion criteria comprised the following aspects:

1. Hearing-impaired older adults with a minimum age of 50 years or a reported mean age of 60 or older, living independently in the community.

Requirements for the study design: Investigation of healthy and/or hearing-impaired older adults in either a randomized control trial (RCT), an experimental–control group design or an old–young comparison with a distinction between older hearing-impaired and non-impaired older adults.

(2) Integration of a dual-task or multitasking.

In this review cognitive-motor Interference (CMI) will be defined as a measure of dual task (DT) performance in comparison to a baseline single task (ST) measurement

Assessment criteria:

1. Investigation of at least one walking or balance task in a DT setting.
2. Assessment of DT performance (ST vs. DT) and/or the dual-task costs.

In order to categorize results across studies so that they were comparable, cognitive tasks were classified according to their modality (e.g., visual, or auditory) and task setting (e.g., stimulus detection vs. stimulus discrimination tasks).

(3) Report of at least one of the main motor outcomes (balance and/or gait) and/or the dual-task costs.

Balance parameters:

- Postural sway (e.g., root mean square of medial–lateral and anterior–posterior amplitude)
- Electromyography (EMG) activity (e.g., peak amplitudes)
- Center of pressure (COP) or Center of Gravity (COG) displacement variables (e.g., total path length, sway velocity, area of ellipse in anterior–posterior or medial–lateral direction)
- Kinematics (e.g., angular velocities of the hip or knee)

Gait parameters (if possible corrected for body height; (cf. Table 2):

**Table 2:** Spatiotemporal gait parameters

Gait – the medical term used to describe the human locomotor movement of walking in healthy people – is simple in terms of execution, but complex in terms of biomechanics and motor control [35]. Within straight forward gait the commonly examined gait variables can be classified into parameters of rhythm (e.g., single and double support time or cadence) and pace (e.g., speed or stride length). According to the framework by Hollmann et al. [35] we define the spatiotemporal gait parameters as follows:	
**Gait parameter**	**Description**
**Pace**	
Gait speed (cm/s or m/s)	Distance traveled divided by the ambulation time; it is commonly expressed in centimeters per second (cm/s) or meters per second (m/s)
Step length (cm)	Distance that one part of the foot travels in front of the same part of the other foot during each step; typically, the distance from initial contact to initial contact, which in healthy gait usually coincides with heel strike
Stride length (cm)	Distance from initial contact of one lower limb to the next initial contact of the same lower limb
**Base of support**	
Step width (cm) or Step width SD (cm)	Lateral distance from heel center of one footprint to the line of progression formed by two consecutive footprints of the opposite foot or the standard deviation of this distance
**Rhythm**	
Cadence (steps/min) or Step time (s)	Number of steps per minute, sometimes referred to as step rate
Stride time (s)	Time elapsed from initial contact of one foot to initial contact of the opposite foot
Swing time (s)	Time elapsed between the initial contacts of two consecutive footfalls of the same foot
Stance time (s)	Weight bearing portion of each gait cycle initiated at heel contact and ending at toe-off of the same foot; stance time is the time elapsed between the initial contact and the last contact of a single footfall
Single support time (s)	Single support occurs when only one foot is in contact with the ground; single support time is the time elapsed between the last contact of the opposite footfall to the initial contact of the next footfall of the same foot
**Phases**	
Swing (% gait cycle (GC))	Swing phase is initiated with toe off and ends with initial contact of the same foot; swing time is the time elapsed between the last contact of the current footfall to the initial contact of the next footfall of the same foot
Stance time (%GC)	Stance time normalized to stride time
Single support (%GC)	Single support time normalized to stride time
Double support (%GC)	Double support time normalized to stride time. The double support time is approximately 20% of the gait cycle during which both feet are in ground contact
Double support time (s)	Double support time occurs when both feet are in contact with the ground simultaneously; double support time is the sum of the time elapsed during two periods of double support in the gait cycle
**Variability**	
Gait speed (%CV)	Coefficient of variation (%CV or %CoV) reflects the variability for each of the parameters; it is the average standard deviation in the gait parameter divided by the average mean of the gait parameter. Higher values indicate a more variable gait pattern.
Step length or width (%CV)	
Step time (%CV)	
Stride length (%CV)	
Stride time or speed (%CV)	
Swing time (%CV)	
Stance time (%CV)

(4) Dual-task costs for all mentioned parameters (e.g., ST-DT/ST*100) and/or the cognitive task performance when single or dual tasking.
(5) Included studies: randomized controlled trials, quasi-randomized controlled trials, cluster-randomized controlled trials, randomized crossover trials, pre- and post-studies, case control studies, cohort studies and cross-sectional studies.

Articles were excluded when:

1. Sample did not match the age requirement and/or contained no hearing-impaired participants.
2. Study design did not include any motor task or dual task.
3. Populations were selected based on a medical condition (e.g., brain injuries, mild cognitive impairment, dementia, multiple sclerosis, Parkinson’s disease) or if the study took place in a care setting.
4. Studies with a secondary analysis of previous reported results in other included studies.
5. Case studies, conference abstracts and qualitative studies.

Two reviewers (BW and AW) searched titles and abstracts to identify all potentially eligible studies meeting the inclusion criteria. Afterwards the two reviewers independently assessed full paper copies of all of the identified potentially eligible studies to determine which of the studies would be included. Any disagreement on inclusion was resolved by discussion and through arbitration by a third reviewer (KL).

### Data extraction and risk of bias

Two reviewers imported references to a table to extract and collate information in three steps:

1. Overview of all includes studies concerning the author, year of release, study design and aims, dual-task type, population with discrimination of hearing impairments/ no hearing impairments and the respective age, the used definition of hearing-impairment, a list of all observed balance or walking parameters, and the results for the relevant comparisons (cf. Table 4)
2. Quality assessment of the included articles based on a customized checklist. This was done with a modified Downs and Black [36] questionnaire by both first authors independently. As the review did not focus exclusively on intervention studies, all quality criteria with respect to randomized controlled trials (e.g., randomization, follow-up periods etc.) were not assessed. Table 3 therefore includes the report of the quality criteria including the following 16 aspects of the Black and Downs scale ( [36]; cf. Table 3). If a quality criterion was described sufficiently, it was rated with a point. Consequently, the maximum quality score is 16 points.
3. For all included studies, the main results were summarized in Table 4. This includes task order, outcome measures used to assess and report the performance of either of the concurrent tasks, and study results.

#### Data items

The data items included the used classification of participants with respect to hearing impairment, walking and balance parameters in single and dual-task conditions. For the walking performance, there is already an agreement as to which outcomes should be measured and reported [e.g., [35, 37]. Therefore, the reported data of walking speed (gait velocity) as well as step length and others like step width are commonly comparable.

In case it was required, the corresponding authors of the included studies were asked to provide additional data to the reported data of the published manuscript. Moreover, the corresponding authors were asked to provide missing data of interest (e.g., if ST vs. DT for baseline conditions were not reported).

#### Data synthesis

We first reported the chosen definition for mild and/or severe hearing impairment. Then, we extracted available data of the comparison of ST and DT or dual-task costs for the hearing-impaired and non-hearing-impaired older adults for each of the outcome variables of interest as a verbal description into Table 4. Available differences between hearing-impaired and non-hearing-impaired older adults were provided.

## Results

**Fig. 1:**
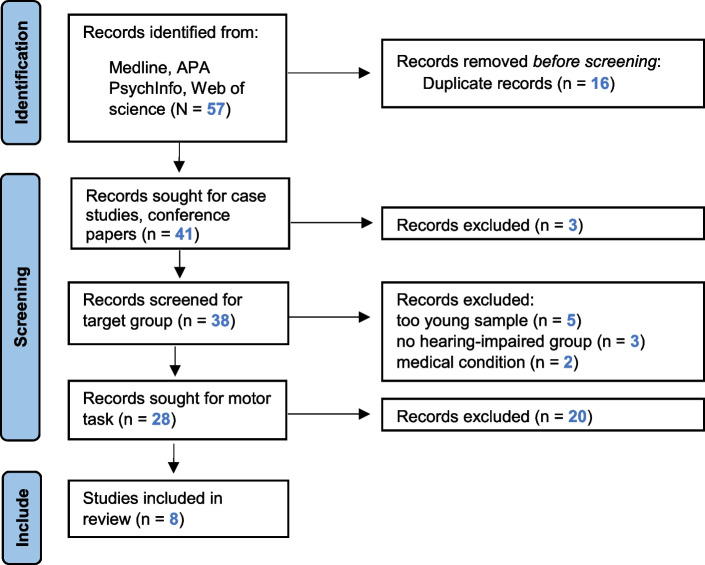
Flow chart of screening stages after initial search

The initial search generated 57 articles including 16 duplicates (Fig. 1) from which a total number of eight studies were integrated into further analysis (cf. Table 4).

Overall, the studies tested *N* = 456 participants (58% women), including 174 healthy older adults and 200 older hearing-impaired persons with different levels of hearing loss. The other 82 participants were young and healthy adults or from other clinical populations. The quality assessment (see Table 3) showed that all eight studies included in this review reached at least nine points and are of high quality.

**Table 3:** Quality assessment

Author	Quality criteria																Sum	Remarks
	**a**	**b**	**c**	**d**	**e**	**f**	**g**	**h**	**i**	**j**	**k**	**l**	**m**	**n**	**o**	**p**		
Bruce et al., 2017 [38]	1	1	1	1	1	1	1	1	1	1	1	1	-	-	1	1	14	c: anthropometric data is missing e: only age, sex reported o: parameter for cognitive abilities missing
Bruce et al., 2019 [30]	1	1	1	1	1	1	1	1	1	1	0	1	-	-	1	0	12	j: only no attrition statement k: small n for age-related hearing loss group, no tests reported whether ANOVA requirements are met m: details are missing n: recruitment was time dependent
Gorecka et al., 2018 [39]	1	1	1	1	1	1	1	1	1	1	1	1	1	-	1	1	15	h: probability values only reported in table o: controlling for hearing acuity
Gorecka et al., 2021 [40]	1	1	1	1	1	1	1	1	1	1	1	1	1	-	1	1	15	c: anthropometric data is missing k: not adjusted to multiple testing, small sample size o: multiple regression of age, sex, education, PTA as covariate
Kowalewski et al., 2018 [41]	1	1	1	1	1	0	0	0	1	1	0	1	-	-	1	0	9	a: introduction deals with falls c: comorbidities are missing f: no post-hoc comparisons g: only in bar plots k: not all statistical values reported o: exclusion of cognitive or sensory impairments
Lau et al., 2016 [42]	1	1	1	1	1	1	1	1	1	1	0	1	1	-	0	0	12	c: anthropometric data is missing k: no correction for multiple comparisons p: small sample size
Wollesen et al., 2018 [29]	1	1	1	1	1	1	1	1	1	1	1	1	1	1	1	0	15	i: 30% of those entering the clinic completed survey and tests n: two testing periods within one year at a similar time of year p: Subgroups of fallers and low SPPB individuals were underpowered when divided by hearing status
Wollesen et al., 2021 [43]	1	1	1	1	1	1	1	0	1	1	1	1	1	1	-	0	13	d: Very brief description provided, plus reference to another paper i: 1 female and 5 male participants o: not applicable p: limitation by small sample size

1 – yes; 0 – no;—– unable to determine; a – Hypothesis/aim/objective clearly described; b – Main outcomes in Introduction or Methods; c – Patient characteristics clearly described; d – Intervention of interest, i.e., dual-task, clearly described; e – Principal confounders described; f – Main findings clearly described; g – estimate of random variability in data for main outcomes; h – Probability values reported for main outcomes; i – Subjects asked to participate were representative of source population; j – Any data dragging clearly described; k – Appropriate statistical tests performed; l – Outcome measures were reliable and valid; m – All participants recruited from the same source population; n – All participants recruited over the same time period; o – Adequate adjustment of confounding; p – Sufficient power to detect treatment effect at significance level of 0.05

*ANOVA* Analysis of variance, *PTA* Pure tone average

Most of the included studies oriented their inclusion criteria for the hearing-impaired group based on the previously published thresholds of the WHO regards mild hearing loss with PTA (0.5-4 kHz) ≥ 25 and < 40 dB. The two Wollesen et al. studies considered instead [43] or in addition [29] moderate hearing loss with PTA (0.5-4 kHz) ≥ 40 dB and < 60 dB and severe hearing loss with PTA (0.5-4 kHz) ≥ 60 dB. Lau et al. [42] included only participants with a threshold PTA (0.5,1,2,3 kHz of both ears) > 25 dB HL and who were experienced hearing aid users. The authors of Kowalewski et al. [41] do not report any PTA threshold but all participants in the hearing loss group had been diagnosed with hearing loss. There were some minor differences in which frequencies were averaged and whether the value for the better, worse or both ears was used for the grouping criteria.

Table 4 presents the main results of all included studies sorted by motor task.

**Table 4:** Included studies sorted by movement task

	**Author**	**Study design**	**Study aims**	**Dual-task type**	**No. of participants**	**Age (y)**	**Definition hearing loss**	**Gait parameters Pace, rhythm, phases, variability**	**Results of relevant comparisons**
**Walking studies**									
1.	Gorecka et al., 2018 [39]	Cross-sectional study	the aim was to evaluate possible asymmetric effects of idchotic listening in a dual-task paradigm during walking overground in right-handed healthy older adults and secondly, to assess the moderating effects of hearing loss on this experimental situation	Walking + audio-spatial stimulus detection (attention; Bergen dichotic listening paradigm)	N Young-Old = 38 (26 female, 12 mild hearing-impaired) N Old-Old = 40 (24 female, 30 mild hearing-impaired)	Young-old: 65.4 ± 2.9 Old-Old: 76.4 ± 4.8	PTA (0.5,1,2, 4 kHz of worse ear) > 24 dB HL	**Pace** Step length (cm) Gait speed (m/s) Stride length (cm) **Base of support** Step width (cm) **Rhythm** n.r **Phases** n.r **Variability** (%CV) Step length (%CV) Gait speed (%CV) Stride length (%CV) Step width (%CV)	**Bilateral gait parameters young-old (YO, 32% hearing-impaired) vs. old-old (OO, 75% hearing-impaired)** **Gait speed** YO > OO **Stride length** YO > OO **Step length (%CV)** YO < OO group effects remained after controlling for hearing ability
2.	Gorecka et al., 2021 [40]	Cross-sectional study	The main goal was to determine whether spontaneous vs. volitional focus of attention evoked quantitative and qualitative impairments on gait in MCI individuals as compared to healthy controls	Walking + audio-spatial stimulus detection (attention; Bergen dichotic listening paradigm)	N OA = 52 (28 female, 28 mild-hearing impaired) N MCI = 43 (23 female)	OA: 70.90 ± 7.35 MCI: 71.19 ± 8.75	PTA (0.5,1,2, 4 kHz of worse ear) > 19.5 dB HL	**Pace** Step length (cm) Gait speed (m/s) **Base of support** Step width (cm) **Rhythm** n.r **Phases** n.r **Variability** (%CV) Step length (%CV) Gait speed (%CV) Step width (%CV)	**Bilateral gait parameters normal hearing vs. hearing impaired** (unpublished additional analysis results provided by Gorecka et al.): **Gait speed** (m/s) normal hearing > hearing impaired (in all conditions) **Step width** (cm) normal hearing < hearing impaired (in all conditions) **Step length (%CV)** normal hearing < hearing impaired (in all conditions) **Gait speed (%CV)** normal hearing < hearing impaired (in all conditions)
3.	Lau et al., 2016 [42]	Cross-sectional study	to investigate the effect of age-related hearing loss (ARHL) on word recognition during multitasking within a setting that is more ecologically valid	Walking + audio-spatial stimulus detection (attention; Bergen dichotic listening paradigm)	N OA = 8 (6 female) N OA hearing loss = 8 (5 female)	OA: 69.9 ± 5.4 OA hearing loss: 73.3 ± 8.4	PTA (0.5,1,2, 3 kHz of both ears) > 25 dB HL and hearing aid users	**Pace** Step length (m) Gait speed (m/s) **Base of support** Step width (m) **Rhythm** Cadence (steps/min) Stride time (s) **Phases** n.r **Variability** Step length (%CV) Gait speed (%CV) Step width(%CV) Cadence (%CV) Stride time (%CV) **Variability in degree (°) / Root mean Square (RMS)** Head angles (°/RMS]) Trunk angles (°/RMS) Head/trunk pitch (°/RMS)	**Gait parameters normal hearing vs. hearing impaired:** **Stride time (%CV)** normal hearing < hearing impaired (in all conditions) **Kinematic Dual-Task Costs:** **Significant Dual-tasks costs differences in both groups:** Mean head pitch; RMS head pitch; RMS trunk pitch **Significant Dual-tasks costs differences only in HL group:** Mean trunk pitch
4.	Wollesen et al., 2021 [43]	Inter-ventional study	to assess the feasibility and acceptability of a multitask training to improve walking performance of older adults with moderate to severe hearing impairment. Moreover, assessing if the program improves walking capacity and multitasking walking performance	Walking + Inhibition (Stroop task)	N OA hearing impairment = 6 (1 female)	OA hearing impairment: 81 ± 6.5	PTA for moderate 41-60 dB HL and severe > 60 dB HL	**Pace** Step length (cm) Gait speed (cm/s) Walking Capacity (m; six-minute walking test [6MWT] distance) **Base of support** n.r **Rhythm** n.r **Phases** Double support time **Variability** n.r	**Post-intervention results** in comparison **to baseline** (pre-intervention): Walking Capacity (m) ↑ Walking speed ↑ (during the dual-cognitive) step length ↑ (in participants who completed all sessions)
5.	Wollesen et al., 2018 [29]	Cross-sectional study	The aim of this study was to identify DT and TT effects on walking speed, step length, and cadence in adults with hearing impairment, previous falls, and physical limitations	Walking + Inhibition (Stroop task)	N Normal Hearing = 21 (10 female) N Mild Impairment = 29 (11 female) N Moderate/ Severe Impairment = 23 (12 female)	Normal Hearing: 64 ± 14 Mild Impairment: 71 ± 10 Moderate/ Severe Impairment: 78 ± 12	mild: PTA (0.5–4 kHz) > 25 and < 40 dBHL, and moderate/severe: PTA(0.5– 4 kHz) ≥ 40 dBHL in the better ear	**Pace** Step length (m) Gait speed (m/s) **Base of support** n.r **Rhythm** Cadence (steps/min) **Phases** n.r **Variability** n.r	**Gait parameters with regards to hearing competence / hearing vs. hearing impaired:** Walking speed and Cadence ↓ (with increased hearing impairment)
**Balance studies**									
1.	Bruce et al., 2019 [30]	Inter-ventional study	compare the efficacy of Simultaneous and Sequential multimodal training intervention formats	Balance + working memory task (n-back)	*N* = 42 (26 females) N OA hearing loss = 13	OA: 68.05 ± 4.65	PTA (0.5,1,2, 3 kHz of both ears) between 25 and 40 dB HL	**Ellipse area** (mm^2^) during computerized dynamic posturography	**Balance- and cognitive performance at baseline:** Groups did not differ in ST at baseline **Post-intervention results** in comparison **to baseline** (pre-intervention) of sequential and simultaneous training on **working memory task**: Sequential OA group ↑ (in comparison to simultaneous OA) Data showed a trend that the ARHL group improved post training regardless of format
2.	Bruce et al., 2017 [38]	Cross-sectional study	investigate the cognitive compensation hypothesis, wherein decreased auditory and motor functioning are compensated by the recruitment of cognitive resources	Balance + working memory task (n-back)	*N* = 87 (60 female) N YA = 29 (25 female) N OA = 26 (20 female) N OA hearing loss = 32 (15 female)	YA: 21.83 ± 3.01 OA: 65.19 ± 3.26 OA hearing loss: 70.75 ± 5.76	PTA (0.5,1,2, 3 kHz of both ears) between 25 and 40 dB HL	**Angular displacement** Ankle Plantarflexion amplitude (postural sway) Hip extension amplitude (postural sway)	**Effect of attentional load in noise condition (dual task noise vs single task noise):** Cognitive accuracy in ARHL ↓ (during dual task noise) **Dual tasks costs** in ARHL ↑ (in comparison to YA)
3.	Kowa-lewski et al., 2018 [41]	Cross-sectional study	aimed to answer two questions: 1) does hearing loss negatively affect the ability to regain balance as reflected by an increased number of steps needed after a perturbation, and 2) do hearing aids reverse this effect and improve balance control, reflected by a decrease in number of steps needed to regain balance	Balance + working memory/ stimulus detection (Bamford-Kowal-Bench Speech-In-Noise (BKB-SIN) test)	N YA = 20 (9 female) N OA = 20 (15 female) N OA hearing loss = 19 (8 or 9 females; 45% out of 19)	YA: 27.2 ± 3.0 OA: 68.7 ± 4.3 OA hearing loss: 73.2 ± 9.1	Hearing loss diagnosis	**Number of steps** to regain balance	**Postural performance with regards to balance parameters during dual task** (BKB-SIN + perturbation): **Number of steps** ↑ (in older adults with hearing loss) **Number of steps on average of all conditions** ↑ (in older adults with hearing loss in comparison to young and older adults with normal hearing) **Auditory performance:** **BKB-SIN scores on average of all conditions ↑** in older adults with hearing loss (indicating worse performance of older adults with hearing loss in comparison to young and older adults with normal hearing)

*6MWT* Six-minute walking test, *ARHL* Age-related hearing loss, *BKB-SIN* Bamford-Kowal-Bench Speech-In-Noise, *CoV/%CV* Coefficient of variation, *DT* Dual task, *OA* Older adults, *OO* Older-old group, *ST* Single task, *TT* Triple task, *YA* Younger adults, *YO* Younger-old group, *MCI* Mild cognitive impairment, *PTA* Pure tone average

Three of the walking studies report reduced gait speed and step length during dual-task compared to single task walking. While in Gorecka et al. [39] hearing loss moderated most of the differences in motor task performance when comparing a younger and an older group of older participants, Gorecka et al. [40] were able to show that most of the walking parameters (except for step width) decreased in performance between ST and DT, however, differences in direction between young and old, as well as for participants with hearing-impairment were not identified or reported in the original paper. On request, the authors contrasted hearing ability and their data showed increased step width and step length variability for participants with mild hearing loss. Wollesen et al. [29] revealed that increased hearing impairment comes along with a decrease in walking speed and cadence. In the treadmill study of Lau et al. [42], significant dual-task costs were found for hearing-impaired participants when investigating the mean trunk pitch.

The three studies investigating cognitive-motor interference with regards to maintaining balance utilized different balance tasks. Bruce et al. [30] applied the computerized dynamic posturography test, Bruce et al. [38] used a perturbation platform and Kowalewski et al. [41] a dual-belt treadmill system, and thus reported a broader range of motor performance measures. Both Bruce et al. studies [30, 38] did not reveal an additional impact of hearing impairment on the balance parameters. In contrast, Kowalewski et al. [41] were able to show that older adults with hearing loss needed more steps to regain their balance after perturbation compared to age-matched and younger controls.

## Discussion

This systematic review aimed to investigate the impact of dual-task performance on gait or balance parameters in older adults with hearing impairments. To answer these questions, we analyzed the definitions of hearing loss, integrated task combinations, and the interaction between hearing loss severity and the cognitive-motor performance in DT task settings for balance and walking tasks. We hypothesized that participants with hearing impairments would show higher decrements in DT performance compared to older adults without hearing impairments. The review identified eight studies that examined DT balance and walking performance in older adults with hearing impairments. These studies differed in their objectives, dual-task setups, and study designs.

### Definitions of hearing loss

Most studies followed the WHO’s previous recommendation for categorizing hearing impairment severity. The downward adjustment of the thresholds by the WHO highlighted that the effects of hearing-impairment manifest already at an earlier stage than previously assumed, underscoring the importance of interventions to address issues starting with mild hearing impairment. Only, Gorecka et al. [40] used on our request the new threshold for mild hearing impairment (PTA (0.5-4kHz) ≥ 20 dB) for the additional analyses provided for this systematic review. One study classified their older participants based on an existing diagnosis of hearing-impairment [41]. Overall in the reported studies, it seemed more like that increasing severity of hearing impairment and a larger sample enabled to reveal the decrements reflected in the motor performance than the chosen classification approach.

### Integrated task combinations of the DT measurements for balance and walking

The studies used different DT settings to examine the performance levels of older adults with hearing impairments. Balance studies integrated working memory tasks (n-back or Bamford-Kowal-Bench Speech-In-Noise test; cf. Table 4) targeting different cognitive processes during motor control. The working memory tasks address a divided attention paradigm focusing on resource allocation (cf. limited resource hypothesis which claims that there is a shared pool of limited resources for both, the cognitive and the motor task [44]. Similarly, walking studies integrated audio-spatial stimulus detection tasks and visual-verbal inhibition tasks (e.g. Stroop), to examine different aspects of cognitive processing during motor control. The audio-spatial stimulus detection tasks refer directly to the potential problem of sensory integration of the hearing information by hearing-impaired older adults, while the visual component of the Stroop tasks is more related to resources needed for gait stability [45]. As a result, the interpretation of DT performance decrements needs to consider the specific task set-ups.

### DT results balance

The DT balance performance showed greater performance decrements in participants with hearing impairments, characterized by a higher number of steps taken to stabilize balance ( [41]; cf. Table 4). These findings suggest that older adults with hearing impairments allocate more effort to motor control processes during DT situations. However, given the limited number of studies and different cognitive and motor task conditions, general conclusions regarding the DT balance abilities in other DT settings of older adults with hearing impairments cannot be drawn from the reported results.

One study compared hearing-impaired older adults with non-hearing-impaired older adults in DT or balance training interventions [30]. Hearing-impaired participants did not show more baseline decrements in their cognitive and balance abilities compared to healthy older adults. Still, they showed training benefits independent of the training regime while the healthy controls benefited more from successive cognitive and motor training. This suggests that the training benefits differ related to hearing performance. Groups with worse hearing might have faced challenges adapting to the different sensory conditions due to the importance of both vision and hearing in balance control [46]. Tailoring the training to individual hearing and motor abilities could enhance its effectiveness for older adults with hearing impairments [cf. [47, 48]. The simultaneous integration of cognitive and motor processes during training may help compensate for performance decrements related to hearing loss, but this concept requires further investigation.

### DT results walking

The DT walking performance of gait parameters addressing mainly pace and variability results (cf. Table 4) suggest a destabilization of gait in participants with hearing impairment, evidenced by decreased gait speed, step length and increased gait variability within the studies. Moreover, these observations were consistent across different secondary tasks or the task settings (e.g., auditory-verbal working memory or visual-verbal inhibition tasks). However, these tasks could also be referred to as executive function tasks (cf. Diamond [49]) which are highly related to activities of daily life [50]. Specifically, participants with hearing impairments had worse baseline walking conditions and higher DT costs (cf. Wollesen et al. [43] compared to Wollesen et al. [29]), making their gait stability comparable to that of fallers and older adults aged 75 and older (cf. Hollmann [35]). This suggests that sensory loss and decreased mobility in this population might lead to gait instability, resembling the gait patterns of much older individuals. These aspects of decreased gait stability were also expressed by the increased gait variability reported by the studies of Gorecka et al. [39, 40] and Lau et al [42]. The overall observed gait destabilization in hearing-impaired individuals may be attributed to the disruption of the auditory feedback mechanisms and changes in the vestibular system, leading to difficulties to locate the head position during the movement. Moreover, the auditory cues from footsteps are relevant in adjusting gait patterns in the environment [13, 51].

Notably, most studies focused on pace-related parameters to describe walking performance. Future research could explore rhythm, phase and base of support parameters to gain a deeper understanding of gait quality within this population. These additional insights, combined with balance performance data might reveal relevant elements for gait and postural stability training. Nevertheless, the study by Wollesen et al. [43] suggested that DT gait performance can benefit from specific training interventions as reported for the balance data. However, the transfer of these benefits to more complex situations (e.g., triple tasks) was not sustained, suggesting the need for longer training periods and individualization of the interventions to improve DT static and dynamic balance performance.

### Recommendations

In summary, this review provided some insights with respect to cognitive-motor interference of older adults with hearing impairments which can be transferred into future DT studies. Firstly, older adults with hearing impairments showed DT decrements within balance and walking tasks. However, according to the mixture of the different task settings, there should be more comprehensive research combining different task complexities and stimulus input conditions for the cognitive as well as the motor task condition. Studies might compare sitting, standing and walking with different forms of cognitive complexities that are relevant for daily activities and related to the reduced ability of sensory integration of this target group (e.g., detection of auditory and visual stimuli, auditory and visual discrimination tasks, tasks including spatial orientation; auditory tasks including background noise etc.).

With respect to conducting future training interventions, the combination of the addressed cognitive-motor-task combination should reflect the real-world conditions in more ecologically valid scenarios. Interventions should focus on simultaneous training tasks to overcome the analyzed destabilizing effects. The exercises should address the combination of vision and hearing related tasks including balance or walking with a specific focus on sensory integration. As previous studies showed, balance and walking should be considered separately with respect to potential DT decrements [52–54]. Therefore, training interventions should address tasks for balance and for walking performance.

### Strengths and limitations

This review integrated high-quality studies published in the last eight years, underscoring the emerging interest in this research area. The main limitation of this review stems from the heterogeneity of secondary tasks and task settings (especially for studies including balance performance), limiting the comparability and generalizability of the results. Calculating DT costs could have solved this problem. However, due to the heterogeneity of reporting, these DT costs were not accessible.

There might also be differences between the processes of motor control if a secondary task involves vision or hearing. Furthermore, the lack of individualization of the secondary task according to the hearing abilities as, e.g., provided within the papers by Bruce et al. [43 and 30] was missing in the other study designs. Finally, the review acknowledged the potential bias arising from seven out of the eight included studies being provided by three research groups. Therefore, it is necessary to conduct additional studies within this area of research to strengthen the evidence base.

## Conclusions

The included studies within this review demonstrated dual task decrements in balance and walking performance for older adults with hearing impairments. These decrements were consistent across DT settings and study designs, highlighting the need for specific interventions to reduce the cognitive-motor interference (CMI) and maintain balance control or walking stability in daily situations that require concurrent cognitive and motor tasks. However, understanding the underlying mechanisms of CMI in this population requires further investigation. Nevertheless, initial evidence suggests that identifying these mechanisms and designing tailored training interventions requires a certain adaptation according to individual hearing and motor abilities as well as to the requirements for activities of daily living.

### Supplementary Information

Supplementary Material 1.

## Data Availability

All papers included in the systematic review are published. The additional analysis by Claudia Rodríguez-Aranda on the data from Gorecka et al. can be requested from them.
