# Supplementary material for: The impact of cognitive-motor interference on balance and gait in hearing-impaired older adults: a systematic review
Source: Eur Rev Aging Phys Act. 2024 Jun 24;21:17. doi: 10.1186/s11556-024-00350-x (PMC11194914; doi:10.1186/s11556-024-00350-x)
Supplement: Supplementary file 1 — Supplementary Material 1. [file 11556_2024_350_MOESM1_ESM.docx]

| Authors | Publication Year | Title | | Exclusion criteria met | |  | Citation |
| --- | --- | --- | --- | --- | --- | --- | --- |
| Buyle et al. | 2021 |  | Functional Gait Can Be Affected by Noise: Effects of Age and Cognitive Function: A Pilot Study |  | no HI |  | Front Neurol. 2021 Feb 9;12:634395. doi: 10.3389/fneur.2021.634395. eCollection 2021. |
| Castro-Chavira et al. | 2021 |  | Effects of dichotic listening on gait domains of healthy older adults during dual-tasking: An exploratory observational study |  | mild HI not separated from HO |  | Human movement science, 75, 102720. doi:10.1016/j.humov.2020.102720 |
| Cho et al. | 2019 |  | Clinical Performance Evaluation of a Personal Sound Amplification Product vs a Basic Hearing Aid and a Premium Hearing Aid |  | no motor task |  | JAMA Otolaryngol Head Neck Surg. 2019 Jun 1;145(6):516-522. doi: 10.1001/jamaoto.2019.0667. |
| Cruz et al. | 2020 |  | The effects of using hearing aids and a frequency modulated system on listening effort among adolescents with hearing loss |  | no motor task |  | Int J Audiol. 2020 Feb;59(2):117-123. doi: 10.1080/14992027.2019.1671992. Epub 2019 Sep 27. |
| Dell'Acqua, Pashler & Stablum | 2003 |  | Multitasking costs in close-head injury patients. A fine-grained analysis |  | no motor task, injury patients |  | Exp Brain Res. 2003 Sep;152(1):29-41. doi: 10.1007/s00221-003-1512-7. Epub 2003 Jun 12. |
| Greters et al. | 2017 |  | Hearing performance as a predictor of postural recovery in cochlear implant users |  | too young |  | Braz J Otorhinolaryngol 2017; 83(1):16–22. doi: 10.1016/j.bjorl.2016.01.002 |
| Gustafson, Ricketts & Picou | 2021 |  | Individual Differences Offer Insight Into Clinical Recommendations for Directional and Remote Microphone Technology Use in Children |  | no motor task, children |  | J Speech Lang Hear Res. 2021 Feb 17;64(2):635-650. doi: 10.1044/2020_JSLHR-20-00281. Epub 2021 Jan 19. |
| Gygi & Shafiro | 2014 |  | Spatial and temporal modifications of multitalker speech can improve speech perception in older adults |  | no motor task |  | Hear Res. 2014 Apr;310:76-86. doi: 10.1016/j.heares.2014.01.009. Epub 2014 Feb 13. |
|  |  |  |  |  |  |  |  |
| Helfer et al. | 2020 |  | Early aging and postural control while listening and responding |  | too young |  | J Acoust Soc Am. 2020 Nov;148(5):3117. doi: 10.1121/10.0002485. |
| Helfer et al. | 2020 |  | Postural Control While Listening in Younger and Middle-Aged Adults |  | too young |  | Ear Hear. 2020 Sep/Oct;41(5):1383-1396. doi: 10.1097/AUD.0000000000000861. |
| Hick & Tharpe | 2002 |  | Listening effort and fatigue in school-age children with and without hearing loss |  | children |  | J Speech Lang Hear Res. 2002 Jun;45(3):573-84. doi: 10.1044/1092-4388(2002/046). |
| Hornsby | 2013 |  | The effects of hearing aid use on listening effort and mental fatigue associated with sustained speech processing demands |  | no motor task |  | Ear Hear. 2013 Sep;34(5):523-34. doi: 10.1097/AUD.0b013e31828003d8. |
| Kokx-Ryan et al. | 2015 |  | Benefits of Nonlinear Frequency Compression in Adult Hearing Aid Users |  | no motor task |  | J Am Acad Audiol. 2015 Nov-Dec;26(10):838-55. doi: 10.3766/jaaa.15022. |
| McGarrigle et al. | 2019 |  | Behavioral Measures of Listening Effort in School-Age Children: Examining the Effects of Signal-to-Noise Ratio, Hearing Loss, and Amplification |  | no motor task, children |  | Ear Hear. 2019 Mar/Apr;40(2):381-392. doi: 10.1097/AUD.0000000000000623. |
| Montero-Odasso et al. | 2020 |  | CCCDTD5 recommendations on early non cognitive markers of dementia: A Canadian consensus |  | review |  | Alzheimers Dement (N Y). 2020 Oct 17;6(1):e12068. doi: 10.1002/trc2.12068. eCollection 2020. |
| Neher, Grimm & Hohmann | 2014 |  | Perceptual consequences of different signal changes due to binaural noise reduction: do hearing loss and working memory capacity play a role? |  | no motor task |  | Ear Hear. 2014 Sep-Oct;35(5):e213-27. doi: 10.1097/AUD.0000000000000054. |
| Pals et al. | 2019 |  | Effects of Additional Low-Pass-Filtered Speech on Listening Effort for Noise-Band-Vocoded Speech in Quiet and in Noise |  | no motor task |  | Ear Hear. 2019 Jan/Feb;40(1):3-17. doi: 10.1097/AUD.0000000000000587. |
| Paraskevoudi, Balci & Vatakis | 2018 |  | "Walking" through the sensory, cognitive, and temporal degradations of healthy aging |  | review |  | New York Academy of Sciences, 10.1111/nyas.13734. Advance online publication. doi: 10.1111/nyas.13734 |
| Picou & Ricketts | 2017 |  | How directional microphones affect speech recognition, listening effort and localisation for listeners with moderate-to-severe hearing loss |  | no motor task |  | Int J Audiol. 2017 Dec;56(12):909-918. doi: 10.1080/14992027.2017.1355074. Epub 2017 Jul 25. |
| Picou & Ricketts | 2018 |  | The relationship between speech recognition, behavioural listening effort, and subjective ratings |  | no motor task |  | Int J Audiol. 2018 Jun;57(6):457-467. doi: 10.1080/14992027.2018.1431696. Epub 2018 Jan 30. |
| Picou & Ricketts | 2014 |  | The effect of changing the secondary task in dual-task paradigms for measuring listening effort |  | no motor task |  | Ear Hear. 2014 Nov-Dec;35(6):611-22. doi: 10.1097/AUD.0000000000000055. |
| Picou, Aspell, & Ricketts | 2014 |  | Potential benefits and limitations of three types of directional processing in hearing aids |  | no motor task |  | Ear Hear. 2014 May-Jun;35(3):339-52. doi: 10.1097/AUD.0000000000000004. |
| Picou, Gordon & Ricketts TA | 2016 |  | The Effects of Noise and Reverberation on Listening Effort in Adults With Normal Hearing |  | no motor task |  | Ear Hear. 2016 Jan-Feb;37(1):1-13. doi: 10.1097/AUD.0000000000000222. |
| Picou, Moore & Ricketts | 2017 |  | The Effects of Directional Processing on Objective and Subjective Listening Effort |  | no motor task |  | J Speech Lang Hear Res. 2017 Jan 1;60(1):199-211. doi: 10.1044/2016_JSLHR-H-15-0416. |
| Picou, Ricketts & Hornsby | 2013 |  | How hearing aids, background noise, and visual cues influence objective listening effort |  | no motor task |  | Ear Hear. 2013 Sep;34(5):e52-64. doi: 10.1097/AUD.0b013e31827f0431. |
| Purdy et al. | 2017 |  | Impact of cognition and noise reduction on speech perception in adults with unilateral cochlear implants |  | cochlear implants |  | Cochlear Implants Int. 2017 May;18(3):162-170. doi: 10.1080/14670100.2017.1299393. Epub 2017 Mar 24. |
| Soylemez & Mujdeci | 2020 |  | Dual-task performance and vestibular functions in individuals with noise induced hearing loss |  | too young |  | Am J Otolaryngol. 2020 Nov-Dec;41(6):102665. doi: 10.1016/j.amjoto.2020.102665. Epub 2020 Aug 11. |
| Suarez & Ferreira | 2019 |  | Role of Auditory Information in Motor Control of the Balance System in Patients with Cochlear Implants |  | spanish, Cochlear |  | Anales de la Facultad de Medicina. 6. 8-24. 10.25184/anfamed2019v6n2a10. |
| Tun, McCoy & Wingfield | 2009 |  | Aging, hearing acuity, and the attentional costs of effortful listening |  | no motor task |  | Psychol Aging. 2009 Sep;24(3):761-6. doi: 10.1037/a0014802. |
| Weatherless, Fedele, Kehring & Letowski | 2013 |  | The Effects of Simulated Hearing Loss on Speech Recognition and Walking Navigation |  | no HI, too young |  | Human factors, 55(2), 285–297. doi: 10.1177/0018720812453465 |
| Wright & Gagné | 2021 |  | Acclimatization to Hearing Aids by Older Adults |  | no motor task |  | Ear Hear. 2021 Jan/Feb;42(1):193-205. doi: 10.1097/AUD.0000000000000913. |
| Wunderlich et al. | 2021 |  | Dual-Task Performance in Hearing-Impaired Older Adults-Study Protocol for a Cross-Sectional Mobile Brain/Body Imaging Study |  | protocol |  | Front Aging Neurosci. 2021 Nov 12;13:773287. doi: 10.3389/fnagi.2021.773287. eCollection 2021. |
| Xia et al. | 2015 |  | Spatial release of cognitive load measured in a dual-task paradigm in normal-hearing and hearing-impaired listeners |  | no motor task |  | J Acoust Soc Am. 2015 Apr;137(4):1888-98. doi: 10.1121/1.4916599. |
